# Probiotics and irritable bowel syndrome

**DOI:** 10.3402/mehd.v23i0.18573

**Published:** 2012-06-18

**Authors:** Riitta Korpela, Leena Niittynen

**Affiliations:** Medical Nutrition Physiology, Pharmacology, Institute of Biomedicine, University of Helsinki, Helsinki, Finland

**Keywords:** IBS, probiotic, irritable bowel syndrome, gastrointestinal symptoms, lactobacilli, bifidobacteria

## Abstract

**Background:**

Irritable bowel syndrome (IBS) is a major cause of abdominal discomfort and gut dysfunction worldwide. It is a poorly understood functional gastrointestinal disorder for which no effective medication is available. It is a benign condition, but its social and economic burden is significant. The symptoms consist of abdominal pain, bloating, flatulence, and irregular bowel movements. Alterations in the intestinal microbiota and mucosal inflammation may contribute to the development of IBS and probiotics could thus relieve the symptoms. This review gives an overview on the existing data on the effects of probiotics on the gastrointestinal symptoms of IBS.

**Methods:**

A PUBMED search was made to review the relevant literature, and additional studies were obtained from the references of the selected articles.

**Results:**

Clinical trials suggest that certain probiotics or combinations of bacteria have beneficial effects on the IBS symptoms. However the heterogeneity of studies, e.g. suboptimal study design, inadequate number of subjects, different doses and vehicles, inadequate length, make it difficult to compare the differences between probiotics and the effect may be strain-specific.

**Conclusions:**

Though evidence is very promising, no general recommendations on the use of probiotics in IBS can be given yet. Further clinical trials and data on the mechanisms of action are needed. Probiotics are considered safe and if future scientific data is able to substantiate their efficacy in IBS, they certainly could be a treatment option in relieving the symptoms in IBS.

Irritable bowel syndrome (IBS) is the most common diagnoses in gastroenterology. It is estimated that approximately 10–20% of the adult population suffers from this syndrome (1). It is a heterogeneous functional disorder that impacts the individual's quality of life as well as the whole society by greatly increasing medical costs. The diagnosis is based on symptoms through the Rome III criteria often following a long medical process of excluding organic diseases (2). The symptoms include abdominal pain, distension, flatulence, and irregular bowel movements (3). It is a condition for which no reliable biological, endoscopic, or radiologic finding nor effective pharmacological treatment is available.

The pathophysiology of IBS is only partly understood, but there is growing evidence of the role of imbalance of the intestinal microbiota, intestinal infections, and a dysfunctional intestinal barrier in the development of IBS and its symptoms [for review see Clarke et al. (4)]. Therefore, the therapeutic potential of probiotics has gathered a lot of interest.

Numerous clinical trials have investigated the effects of probiotics in patients with IBS, but the evidence is not yet consistent due to small number of subjects, variability in study design, heterogeneity of probiotic strain, dose and treatment duration, and patient characteristics in the clinical studies [for review, see Lee and Bak (5)]. However, some of the probiotics or their combinations have shown promising beneficial effects. The most commonly studied probiotics in IBS are lactobacilli and bifidobacteria, but findings from one strain should not be extrapolated to other strains as stressed by FAO/WHO joint expert report (6).

The aim of this summary is to give an overview based on existing evidence on the effects of probiotics in the treatment of symptoms in IBS.

## Clinical trials on single probiotic strains

Summary of randomized controlled trials on the effects of probiotics in the treatment on IBS is shown in Table 1.

**Table 1:** Randomized controlled trials of probiotics on IBS

Probiotic	Number of subjects	Duration	Result	Reference
*B. infantis* 35624	77	8 weeks	Pain, IBS score, bowel movement difficulty ↓	7
	362	4 weeks	Abdominal pain, IBS score, distension, incomplete evacuation, straining, flatulence ↓	8
*B. animalis* DM 173010	274	6 weeks	Stool frequency in subjects with <3 stools/week ↑	10
*B. bifidum* MIMBb75	122	4 weeks	IBS symptoms, pain, discomfort, distension, bloating, urgency, digestive disorder ↓	9
*L. plantarium* 299V	60	4 weeks	Flatulence ↓	11
	20	4 weeks	IBS score, abdominal pain ↓	13
	12	4 + 4 weeks	↔	18
*L. rhamnosus* GG	24	8 + 8 weeks	↔	12
	50 children	6 weeks	Abdominal distention ↓	14
	104 children	4 weeks	Treatment success ↑; abdominal pain frequency ↓	15
	141 children	12 weeks	Treatment success ↑; abdominal pain ↓	17
*L. acidophilus*-SDC 2012, 2013	40	4 weeks	Treatment success ↑; abdominal pain and discomfort ↓	16
*L. acidophilus*	61	2 weeks	↔	19
*L. reuteri* ATCC 55730	54	6 months	↔	20
*L. plantarum* MF1298	16	3 + 3 weeks	IBS sum score ↑	21
*S. boulardii*	67	4 weeks	↔	22
	35	30 days	↔	23
*Streptococcus faecium*	54	4 weeks	Clinical improvement ↑	24

### Bifidobacteria

Most randomized, placebo-controlled studies have suggested that *Bifidobacterium* have beneficial effects on IBS symptoms (7–9). In a trial with 77 IBS patients, ingestion of *Bifidobacterium infantis* 35624 for 8 weeks reduced pain, IBS score, and bowel movement difficulty (7). In another study, 362 women with all subtypes of IBS were treated with three different doses of *B. infantis* 35624 (10^6^, 10^8^, and 10^10^ cfu) for 4 weeks. It was reported that only the dose of 10^8^ cfu was significantly superior to placebo in reducing abdominal pain, IBS score, distension, incomplete evacuation, straining, and flatulence (8).

In a multicenter, placebo-controlled trial with 274 adults with constipation-predominant IBS, a fermented milk containing *Bifidobacterium animalis* DM 173010 showed beneficial effect on health-related quality of life during the 6-week study period. In addition, stool frequency increased in the subjects with less than three bowel movements per day. However, there were no other statistically significant differences in the symptoms of IBS between the placebo and probiotic groups (10).

Gugliametti et al. (9) investigated the effects of *Bifidobacterium bifidum* MIMBb75 on the severity of IBS in 122 patients. After the treatment for 4 weeks, probiotics reduced the global assessment of IBS symptoms, improved pain, discomfort, distension, bloating, urgency, and digestive disorder. In addition, *B. bifidum* MIMBb75 group showed a significant improvement in the quality of life. Overall, responder rates were 57% in the probiotic group and 21% in the placebo group (*p*<0.0001).

### Lactobacilli

Placebo-controlled trials with partly contradictory results have investigated the effects of *Lactobacillus* species on IBS symptoms (11–17). *Lactobacillus plantarium* 299V has shown beneficial effects in two controlled trials (11, 13). Nobaek et al. (11) investigated the effects of *L. plantarium* 299V in 60 patients with IBS. During the 4-week treatment period, flatulence reduced rapidly and significantly in the probiotic *versus* the placebo group, but there were no differences in the reduction of abdominal pain between the groups. In another 4-week study, all 20 patients treated with *L. plantarium* 299V reported reduction of their abdominal pain as compared to 11/20 patients from a placebo group (*p*=0.0012). IBS symptoms were improved in 95% of patients in the *L. plantarium* 299V group *versus* 15% of patients in the placebo group (*p*<0.0001) (13). On the contrary, one 4-week study failed to show any beneficial effect of *L. plantarium* 299V (18).

Several controlled trials have investigated the effects of *Lactobacillus rhamnosus* GG on the symptoms of IBS in adults and in children (12, 14, 15, 17). These studies have suggested that *L. rhamnosus* GG is not effective in the treatment of common intestinal discomfort in adults, but it may offer some help to children suffering from IBS. Francavilla et al. (17) investigated the effect of *L. rhamnosus* GG in 141 children with IBS or functional pain. Compared with the baseline symptoms, *L. rhamnosus* GG, but not placebo, significantly reduced both the frequency (*p*<0.01) and severity (*p*<0.01) of abdominal pain. At the end of the 12-week treatment period, treatment success was achieved in 48 children in the *L. rhamnosus* GG group compared with 37 children in the placebo group (*p*<0.03), and this difference still was present at the end of follow-up without microbial treatment (*p*<0.03). In another trial, 104 children who fulfilled the Rome II criteria for IBS, functional dyspepsia, or functional abdominal pain received *L. rhamnosus* GG or placebo for 4 weeks. Those children in the *L. rhamnosus* GG group were more likely to have treatment success (33 vs. 5%) and reduced frequency of pain (*p*=0.02) compared to those in the placebo group, but there were no differences in pain severity between the groups (15). In a 6-week study with 64 children with IBS, *L. rhamnosus* GG was not superior to placebo in the treatment of abdominal pain, but it relieved abdominal distention (14).

In a Korean study, ingestion of *Lactobacillus acidophilus*-SDC 2012, 2013 for 4 weeks reduced score for abdominal pain and discomfort compared to the baseline and led to better treatment success than placebo (16). On the other hand, some studies have failed to show any beneficial effect of *Lactobacillus* compared with the controls (12, 18–20), and it has also been reported that some *Lactobacillus* species may even have unfavorable effects in patients with IBS. Ligaarden et al. (21) investigated the effects of *L. plantarum* MF1298 in 16 subjects with IBS in a placebo-controlled double-blind crossover trial. Treatment periods lasted for 3 weeks and wash-out period for 4 weeks. Thirteen patients (81%) preferred placebo to probiotic treatment. In the probiotic period, IBS sum score was 6.44 compared to 5.35 in the placebo period (*p*=0.01).

These results suggest that lactobacilli may be a valuable alternative in the treatment of IBS, but the effect is most probably strain specific.

### Saccharomyces

According to two published trials, *Saccharomyces boulardii* seems not to be effective in the treatment of IBS symptoms in adults. Choi et al. (22) evaluated the effects of *S. boulardii* on quality of life and symptoms in patients (n = 67) with diarrhea-predominant IBS or mixed-type IBS. After the 4-week study period, *S. boulardii* improved quality of life better than placebo but had no effect on the symptoms of IBS. Kabir et al. (23) reported that treatment with *S. boulardii* for 30 days in diarrhea-predominant IBS patients did not result in any improvement in symptom score or personal and professional life.

### Streptococcus

In a Danish study, a comparison of a culture of *Streptococcus faecium* and placebo was carried out in 54 patients with IBS. After the 4-week treatment period, 81% of the active and 41% of the placebo-treated patients had improved according to the physicians’ overall assessment (*p*=0.002) (24).

## Clinical trials on multispecies probiotic mixture

Because of the diverse nature of IBS, it has been suggested that probiotic combinations could be more effective than single strains in this particular disease. Several multispecies probiotics have shown beneficial effects in the treatment of IBS symptoms in clinical controlled trials (Table 2). Two studies with *L. rhamnosus* GG in a combination with *L. rhamnosus* LC705, *Propionibacterium freudenreichii* spp. *shermanii* JS and *Bifidobacterium breve* Bb99 or *B. animalis* spp. *lactis* Bb12 found significant reduction in IBS symptoms (25, 26). In the first study (25), 103 patients with IBS took part in the 6-month, randomized, double-blind, placebo-controlled study. At the end of the study, median reduction in the symptom score was 42% in the probiotic group and 6% in the placebo group. In the second study, 86 patients with IBS received *L. rhamnosus* GG in the combination or placebo for 5 months, and the IBS score decreased 37% in the probiotic group and 9% in the placebo group (26).

**Table 2:** Randomized controlled trials of probiotic mixtures on IBS

Probiotic mixture	Number of subjects	Duration	Result	Reference
*L. rhamnosus* GG combination^a^	103	6 months	IBS score ↓	25
	86	5 months	IBS score ↓	26
VSL#3^b^	25	8 weeks	Bloating ↓	27
	48	4 weeks	Flatulence, colonic transit ↓	28
	59 children	6 weeks	IBS symptoms ↓	29
*L. plantarum* LP 01 + *B. breve* BR 03 or *L. plantarum* LP 01 + *L. acidophilus* LA 02	70	4 weeks	IBS symptoms, pain ↓	30
*L. acidophilus* CUL60 + *L. acidophilus* CUL21 + *B. lactis* CUL34 + *B. bifidum* CUL20	52	8 weeks	IBS symptoms, pain ↓	31
*B. bifidum* BGN4 + *B. lactis* AD011 + *L. acidophilus* AD031 + *L. casei* IBS041	70	8 weeks	Abdominal pain ↓	32
*B. longum* LA 101 + *L. acidophilus* LA 102 + *L. lactis* LA 103 + *S. thermophilus* LA	100	4 weeks	↔	33

a LGG^®^ combination = *L. rhamnosus* GG, *L. rhamnosus* LC705, *P. freudenreichii* spp. *shermanii* JS, and *B. breve* Bb99 or *B. animalis* spp. *lactis* Bb12.

b VSL#3 = *B. longum, B. infantis, B. breve, L. acidophilus, L. casei, L. bulgaricus, L. plantarum,* and *S. salivarius subsp. thermophilus.*

Three studies have investigated the effects of VSL#3 (a mixture of *B. longum*, *B. infantis*, *B. breve*, *L. acidophilus*, *L. casei*, *L. bulgaricus*, *L. plantarum*, and *S. salivarius* subsp. *thermophilus*) in patients with IBS (27–29). In the first randomized study, 25 patients with diarrhea-predominant IBS received VSL#3 powder or placebo twice daily for 8 weeks. VSL#3 reduced abdominal bloating but had no effect on gastrointestinal or colonic transit (27). In the second study, 48 patients with IBS were randomized in a parallel group, double-blind design to placebo or VSL#3 for 8 weeks. VSL#3 reduced flatulence and retarded colonic transit without altering bowel function (28). Also, in children affected by IBS, VSL#3 has been more effective than placebo in ameliorating symptoms and improving the quality of life during the treatment period of 6 weeks (29).

An Italian study compared the effects of mixtures containing *L. plantarum* LP 01 and *B. breve* BR 03 or *L. plantarum* LP 01 and *L. acidophilus* LA 02, and placebo in 70 adults with IBS. Pain score decreased significantly in the probiotic groups compared to the placebo (45 and 49 vs. 29.5%) after 28 days. Similarly, the severity of IBS symptoms decreased significantly after the use of probiotics (56 and 55.6 vs. 14.4%) (30).

Williams et al. (31) investigated the effects of a probiotic preparation comprising *L. acidophilus* CUL60 and CUL21, *B. lactis* CUL34, and *B. bifidum* CUL20 in 52 IBS patients. Over the 8-week intervention period, a significantly greater improvement in the Symptom Severity Score of IBS and in scores for quality of life, days with pain, and satisfaction with bowel habit was observed in the probiotic group than in the placebo group. In a Korean study, treatment with *B. bifidum* BGN4, *B. lactis* AD011, *L. acidophilus* AD031, and *L. casei* IBS041 for 8 weeks reduced abdominal pain significantly compared to placebo in adults with IBS (32). On the contrary, a probiotic combination with *B. longum* LA 101, *L. acidophilus* LA 102, *L. lactis* LA 103, and *S. thermophilus* LA 104 for 4 weeks was not significantly superior to the placebo in relieving symptoms of IBS (33).

## Meta-analyses and reviews

Several reviews (e.g. 4, 5, 34–36) and two meta-analyses (37, 38) have been published to demonstrate the data regarding the use of probiotics in the treatment of IBS.

McFarland and Dublin (37) connected the results of 20 randomized, placebo-controlled, blinded trials of probiotics for the treatment of IBS. In the meta-analysis, probiotic use was associated with improvement in global IBS symptoms (RR_pooled_=0.77) and less abdominal pain (RR_pooled_=0.78). Too few studies reported data on other IBS symptoms to allow estimation of a pooled relative risk. It was suggested that probiotics may be beneficial in the treatment of IBS, but more prolonged studies are needed.

Hoveyda et al. (38) included 14 English, randomized, placebo-controlled studies to their meta-analysis. The length of the studies varied from 4 to 26 weeks, and different participants and doses of probiotics or probiotic mixtures were used. Combined data suggested a modest improvement in IBS symptoms after the treatment with probiotics. Seven studies reported a statistically significant improvement on abdominal pain (OR = 2.88), five studies reported a statistically significant improvement on flatulence (OR = 2.31), and four studies reported a statistically significant improvement on the symptoms of bloating (OR = 1.75). However, when using continuous outcomes reported on non-standard scales, the improvements in these symptoms were not statistically significant. It was concluded that probiotics may have a role in alleviating some symptoms of IBS, but the optimal type and dose of probiotics and especially the subgroups of patients who are likely to benefit the most remain to be clarified.

Even if meta-analysis in the area of probiotics do not make sense as the active compounds (i.e. the probiotic) are different and their mechanism may differ, one can conclude that they show much promise for the use of probiotics in the treatment of IBS.

Recently, Clarke (4) concluded in his comprehensive review that progress in the field requires adequately powered long-term multicenter trials and the embracing of bench to bedside approaches. According to his conclusion on a evaluation of 42 trials examining the efficacy of lactobacilli in IBS, there is much promise for the use of lactic acid bacteria in the treatment if IBS. Of the 42 trials, 34 reported beneficial effects in at least one of the end points or symptoms examined even if tremendous variation in both the magnitude of effect and the choice of outcomes existed.

Moayyedi et al. (34) published an interesting systematic analysis on the randomized controlled trials testing various probiotics in patients with IBS. They show not only that many trials are of good quality and that positive results were obtained with some strains but also that the studies are heterogenous and that there may be a publication bias.

Brenner et al. (35) conclude in their systematic review that most RCTs about the utility of probiotics in IBS have not used an appropriate study designing and do not adequately report adverse effects. Future studies should follow Rome III criteria recommendations for appropriate design of an RTC.

## Possible mechanisms

Numerous studies have investigated the possible mechanisms behind the beneficial effect of probiotics on IBS symptoms. Several mechanisms, such as altering intestinal luminal environment, maintenance of mucosal barrier function, and modulation of immune system, may explain the reduction of IBS symptoms after the treatment with probiotics [for review, see Lee and Bak (5)].

Alteration of the gut microbiota is related to the pathogenesis of IBS [for review, see Lee and Bak (5)]. No single abnormality in the microbiota of IBS patients has been shown, but several studies have reported alterations in the bacterial composition and stability in IBS (39–42).

Clinical studies have shown that probiotics can alter the gut microbiota with improvements of symptoms of IBS (11, 26, 43). It has also been reported that ingestion of probiotic mixture containing *L. rhamnosus* GG, *L. rhamnosus* LC705, *P. freudenreichii* spp. *shermanii* JS, and *B. animalis* spp. *lactis* Bb12 affected IBS-associated fecal bacterial phylotypes (44). On the contrary, another probiotic mixture VSL#3, which have reduced the symptoms of IBS (27, 28), had no effect on gut microbiota (45). Probiotics could influence symptoms directly through balancing the microbiota and thus normalizing an aberrant gas production.

Elevated intestinal permeability has been documented in IBS patients, and there is growing evidence that disturbances of barrier function may play a role in the development of IBS. In experimental studies, probiotics have improved barrier function [for review, see Lee and Bak (5)]. In a clinical study, *L. rhamnosus* GG normalized the increased intestinal permeability of children with IBS (17).

Dysregulation of immunity can contribute to the pathogenesis of several diseases including IBS. Probiotics have been shown to have various effects on the immune system, and thus, they may be beneficial in the treatment of IBS [for review, see Lee and Bak (5)]. O'Mahony et al. (7) noticed that patients with IBS have abnormal ratio of IL-10/IL-12, which is an indicator of a proinflammatory state. This ratio was normalized after 8 weeks treatment with *B. infantis* 35624. These results suggest that probiotics may have an immune-modulating role in the treatment of IBS.

An inflammatory component seems also to be one possible factor in IBS, especially in so called postinfectious IBS (46). Also, animal models clearly suggest that inflammation could contribute to the symptoms of IBS, since there seems to be a causal relationship between mucosal inflammation and altered sensory-motor function.

It has also been suggested that the role of visceral sensitivity and the dysfunction of brain-gut axis are important factors in the development of IBS symptoms (47, 48). *L. acidophilus* NCFM was able to increase the visceral pain threshold in rats and to upregulate µ-opioid and cannabinoid receptors in colonic epithelial cells and in the colonic epithelium of rodents (49).

As listed above, numerous mechanisms are possible in the action of probiotics in IBS. A probiotic contains thousands of genes, which may potentially influence the clinical effects as was pointed out by Marteau (50). New discoveries will hopefully have an impact on the understanding on intestinal motility and visceral sensitivity and thus the mode of action of probiotics in the treatment of intestinal disorders.

## Conclusions

There already exists rationale behind the use of probiotics in the treatment of IBS although solid strain-specific efficacy and emerging data identifying potential mechanism is needed. Careful trial design, strain selection and blinding, adequate sample size and trial length, optimum dosage, safety, and long-term tolerability must be considered when designing and evaluating clinical studies.
